# Study on the Non-Target Metabolomics Effects of Tylosin on *Pasteurella multocida*

**DOI:** 10.3390/vetsci13040386

**Published:** 2026-04-16

**Authors:** Ting Zhang, Junhao Xiang, Yaoxin Tang, Xiubo Li, Yiming Liu

**Affiliations:** 1 National Feed Drug Reference Laboratories, Feed Research Institute, Chinese Academy of Agricultural Sciences, Beijing 100081, China; 18793598414@163.com (T.Z.); jhxiang2000@126.com (J.X.); 821012410486@caas.cn (Y.T.); lixiubo@caas.cn (X.L.); 2 Key Laboratory of Animal Antimicrobial Resistance Surveillance, Ministry of Agriculture and Rural Affairs, Feed Research Institute, Chinese Academy of Agricultural Sciences, Beijing 100081, China

**Keywords:** tylosin, *Pasteurella multocida*, non-target metabolomics, antimicrobial resistance genes, One Health

## Abstract

*Pasteurella multocida* (*P. multocida*) is not only the core pathogen of bovine respiratory disease (BRDC) but also a significant zoonotic agent, posing a dual threat to global animal husbandry and public health. This study utilized untargeted metabolomics to systematically dissect the metabolic regulatory network of *P. multocida* in response to tylosin within a One Health framework. The results revealed significant “defense–growth” metabolic reprogramming: activation of amino sugar and nucleotide sugar pathways (e.g., CDP-glucose) indicated cell wall remodeling, while directional shifts in the phenylalanine–tyrosine network directed flux toward defensive secondary metabolites. Concurrently, amino acid disorders and the overactivation of the ABC transporter system exacerbated an internal energy crisis, characterized by a shift from respiration to glycolysis, ATP depletion, and ROS accumulation. SEM observations confirmed membrane integrity disruption and cytoplasmic leakage. Crucially, this metabolic stress and the transition into a “persister-like” dormant state are closely linked to the adaptive expression of antimicrobial resistance (AMR) genes. Under the selective pressure of tylosin, these metabolic perturbations may facilitate the emergence and horizontal transfer of resistance determinants, which can circulate through the animal–human–environment interface. By revealing the metabolic physiological basis of tylosin’s action and its role in inducing bacterial tolerance, this study provides critical theoretical insights for antimicrobial stewardship, aiming to mitigate the risk of AMR transmission and preserve the efficacy of macrolides for both veterinary and human medicine.

## Simple Summary

*Pasteurella multocida* is a major pathogen responsible for respiratory diseases in livestock and can also infect humans, making it a significant concern for both animal welfare and public health. Tylosin is a widely used antibiotic in veterinary medicine, but its extensive use raises concerns about the development of antimicrobial resistance (AMR). In this study, we investigated how tylosin affects the internal chemistry (metabolism) of *P. multocida*. We investigated how tylosin treatment alters the metabolic profile of *P. multocida*. We discovered that tylosin induces significant metabolic stress, forcing the bacteria to shift their metabolic pathways, which leads to cell damage and a “dormant” state. Importantly, we hypothesize that this metabolic remodeling may create a permissive environment for bacterial survival and the potential acquisition of resistance genes. which can spread between animals, humans, and the environment. By understanding these metabolic weak spots, our research supports the One Health initiative by providing a scientific basis for better antibiotic use, helping to preserve the effectiveness of these drugs and reduce the risk of resistance spreading to human medicine.

## 1. Introduction

*Pasteurella multocida* (*P. multocida*) is a primary pathogen responsible for bovine respiratory disease complex (BRDC) and hemorrhagic septicemia across a variety of domestic animals [1,2]. Beyond its impact on the livestock industry, it is a significant zoonotic pathogen that poses a threat to public health through direct contact and environmental vectors [3,4]. Tylosin is a 16-membered macrolide antibiotic widely used in veterinary clinics for livestock and poultry. While *P. multocida* is traditionally recognized as a core pathogen of BRDC, it is also a major etiological agent in other significant economic diseases, including porcine respiratory disease complex (PRDC) [5], fowl cholera in poultry [6], and respiratory infections in dogs. Tylosin was specifically selected for this investigation due to its widespread application in veterinary medicine for treating these diverse respiratory infections. Given its role as a key therapeutic agent for food-producing animals, understanding its precise interaction with *P. multocida*—a pathogen with significant zoonotic potential [3]—is critical for managing clinical outcomes and limiting the development of antimicrobial resistance. Within the global “One Health” framework, an in-depth analysis of its antibacterial mechanism is essential for optimizing dosage regimens, minimizing environmental drug residues, and curbing the dissemination of antimicrobial resistance (AMR) genes in the ecosystem [7,8].

Metabolomics, as an important terminal link in systems biology, can reflect real-time metabolic phenotype changes in cells under drug stress. Although the classical mechanism of action of tylosin is to bind to the 50S ribosomal subunit to inhibit protein synthesis [9], specifically by blocking the function of the peptidyl transferase center (PTC) by binding to the 23S rRNA of the bacterial 50S ribosomal subunit [10,11,12], an increasing number of studies have shown that the lethal effect of antibiotics is closely related to the endogenous metabolic state of bacteria. Lobritz et al.’s research indicates that the bacteriostatic efficacy of antibiotics has an inherent logical connection with the cellular respiration level of bacteria [13]. This study suggests that antibacterial drugs induce abnormal acceleration or disorder of the respiratory chain, leading to excessive consumption of metabolic substrates and a burst of reactive oxygen species (ROS). However, previous studies on tylosin have mostly focused on structural interactions with the ribosome and the molecular epidemiology of resistance genes (such as erm and mef) [2,14]. For facultative anaerobic Gram-negative bacteria like *P. multocida*, tylosin treatment may interfere with its rapid growth metabolism mode (r-selection metabolism), triggering metabolic dormancy or persistence [15,16]. However, there is a lack of systematic understanding of the global metabolic reprogramming it causes. Therefore, exploring the balance between the antibacterial mechanism and oxidative stress of *P. multocida* under tylosin treatment is of crucial research significance for understanding its deep bacteriostatic mechanism from a metabolomics perspective.

This study employs metabolomics to elucidate the inhibitory mechanism of tylosin against *P. multocida*. By identifying key metabolic pathways and biomarkers, it provides a theoretical basis for understanding drug action and designing next-generation ribosomal-targeting derivatives. Under the “One Health” framework, these insights facilitate precision dosing to mitigate antimicrobial resistance and environmental footprints, fostering synergistic health for humans, animals, and ecosystems.

## 2. Materials and Methods

### 2.1. Strains and Reagents

The standard strain of *Pasteurella multocida* (CVCC 393) was purchased and preserved by our laboratory. The bovine brain heart infusion medium (BHI) culture medium was purchased from Qingdao Haibo Biotechnology (Qingdao, China), and the ATP and ROS kits were purchased from Beyotime Biotechnology Company (Shanghai, China). PBS buffer and SOD were purchased from Solebao Biotechnology Company (Beijing, China).

### 2.2. Bacteriostatic Growth Curve

*P. multocida* was cultured to the logarithmic growth phase, and the concentration was adjusted to about 10^6^ CFU/mL. A drug group and a blank growth control group were set up, and different tylosin concentration groups were established to measure the bacteriostatic curve for each concentration. The growth control group contained only culture medium and bacterial liquid. Both the drug and bacterial liquid were prepared using BHI. Drugs at each concentration were mixed with 1 × 10^6^ CFU/mL bacterial liquid to obtain mixtures containing drugs and bacterial liquid at 0.5 × MIC, MIC, and 2 × MIC concentrations. The mixtures were cultured at 37 °C with constant temperature shaking. The culture was taken out at 0, 2, 4, 6, 8, 12, and 24 h, and the absorbance value at OD_600nm_ was measured. Set up three repetitions.

### 2.3. Time-Kill Kinetics Assay

The antibacterial activity of tylosin against bovine *P. multocida* was evaluated using time–kill kinetic assays. The experiment first required preparing *P. multocida* (1 × 10^6^ CFU/mL) in the logarithmic growth phase and setting different concentrations of tylosin (0.5 × MIC, MIC, and 2 × MIC) along with a control group according to its MIC value. The bacterial liquid collected at 0, 2, 4, 6, 8, 10, and 24 h needs to be gradient diluted and spread on BHI solid medium. After incubation, the colony-forming units (CFU/mL) were counted. Finally, log10 (CFU/mL) was plotted against time (h) to form the killing curve of the drug against the strain.

### 2.4. Scanning Electron Microscopy (SEM) Analysis

Scanning electron microscopy (SEM) is an observation method between transmission electron microscopy and light microscopy. *P. multocida* was cultured to the logarithmic growth phase, and the concentration was adjusted to about 10^6^ CFU/mL and treated with tylosin (2 × MIC) for 6 h. The cells were collected by centrifugation (8000× *g*, 4 °C, 10 min) and fixed with electron microscope fixative. The bacterial precipitates were fixed with 2.5% glutaraldehyde at 4 °C overnight, then dehydrated using a series of ethanol concentrations (30%, 50%, 70%, 80%, 90%, 95%). Each concentration was washed twice for 15 min. This was followed by dehydration with 100% ethanol twice for 30 min each. Next, ethanol was replaced twice with 100% tert-butyl alcohol for 30 min. After freeze-drying, the samples were sprayed with gold and then placed under a scanning electron microscope.

### 2.5. Metabolomic Analysis

Given that tylosin is traditionally characterized as a bacteriostatic agent, our experiments focused on sub-lethal concentrations designed to induce metabolic reprogramming without achieving a total antibacterial effect. The main steps of metabolite extraction include adding an appropriate amount of extraction solution and magnetic beads for grinding and ultrasonic treatment. After standing for centrifugation, the supernatant was taken for vacuum drying. Then, an appropriate amount of extraction solution was added for reconstitution and detection on the machine. A Waters Acquity I-Class PLUS ultra-high-performance liquid chromatography system was connected in series with a Waters Xevo G2-XS QTOF high-resolution mass spectrometerfrom Waters Corporation (Milford, Massachusetts, USA). Sample detection was analyzed according to the corresponding parameters. The raw data collected using MassLynx V4.2 were used for peak extraction, peak alignment, and other data processing operations through Progenesis QI V3.0 software. The Progenesis QI software was used for identification based on the online METLIN database, public databases, and self-built libraries. At the same time, theoretical fragment identification was performed. Quality control (QC) samples were prepared by mixing equal aliquots of all experimental samples. To monitor instrument stability and data reproducibility, QC samples were injected at regular intervals throughout the analytical sequence. Data quality was validated by ensuring that the relative standard deviation (RSD) for detected metabolites in QC samples was <20%. Biomark was responsible for technical service support.

### 2.6. Reactive Oxygen Species (ROS) Determination

*P. multocida* was cultured to mid-logarithmic phase, and bacterial cells were harvested by centrifugation (8000× *g*, 4 °C, 10 min). The cell pellet was washed twice with phosphate-buffered saline (PBS, pH 7.4) to remove residual culture medium. Subsequently, cells were incubated with 10 µM 2′,7′-dichlorodihydrofluorescein diacetate (DCFH-DA) in the dark at 37 °C for 20 min to facilitate intracellular probe uptake and enzymatic hydrolysis; intracellular esterases cleave DCFH-DA to DCFH, which is subsequently oxidized by reactive oxygen species (ROS) to the highly fluorescent compound 2′,7′-dichlorofluorescein (DCF). The unincorporated probe was removed by two additional PBS washes. Cells were then exposed to tylosin at a concentration of 2× minimum inhibitory concentration (2 × MIC); a vehicle control group (treated with an equivalent volume of solvent only) was included in parallel. Both experimental and control groups were incubated in the dark at 37 °C for 2 h. Fluorescence intensity was quantified using a microplate reader with excitation at 488 nm and emission detection at 525 nm. Intracellular ROS levels were assessed by comparing the mean fluorescence intensity between the tylosin-treated and vehicle control groups.

### 2.7. ATP Determination

*P. multocida* was cultured to the logarithmic growth phase, treated with tylosin (2 × MIC), and a solvent-only control was set up. Incubation was carried out at the present point for 6 h. Subsequently, the bacterial precipitates were collected from each group, and lysozyme (final concentration 1 mg/mL) was added. After incubation at 37 °C for 30 min, an ice bath ultrasound was performed (power 200 W, working for 3 s, intermittent for 5 s, total time 3 min). ATP extract was rapidly added for lysis to release intracellular ATP, and then the samples were centrifuged (10,000× *g*, 4 °C). Take the supernatant after 2 min and then thoroughly mix the sample with the firefly luciferase reaction reagent. Use a luminometer to measure its luminescence intensity (RLU) and finally calculate the ATP concentration in the sample based on the ATP standard curve.

### 2.8. SOD Determination

Culture *P. multocida* to the logarithmic growth phase, adjust the cell density to approximately 10^8^ CFU/mL, harvest bacterial cells via centrifugation (8000× *g*, 4 °C, 10 min), and treat with tylosin at a concentration of 2× minimum inhibitory concentration (2 × MIC). Include a vehicle control group treated with solvent only. Incubate all cultures at 37 °C for 6 h. Subsequently, prepare crude intracellular enzyme extracts by ultrasonic disruption and cell lysis. Following the manufacturer’s instructions for the superoxide dismutase (SOD) assay kit, mix each sample with the designated reaction mixture and incubate under specified conditions. Finally, measure the absorbance at 450 nm.

### 2.9. Data Processing and Statistics

The data were expressed as mean ± standard deviation (SD). Statistical analyses were performed using GraphPad Prism 9.0. Differences between groups were analyzed using an unpaired Student’s *t*-test. *p*-values less than 0.05 were considered statistically significant (* *p* < 0.05, ** *p* < 0.01, *** *p* < 0.001).

Metabolomic data analysis: After normalizing the original peak area information by the total peak area, follow-up analysis was performed. Principal component analysis and Spearman correlation analysis were used to judge the repeatability of samples within groups and the quality control samples. Identified compounds were searched for classification and pathway information in the KEGG, HMDB, and LipidMaps databases. According to the grouping information, calculate and compare the difference multiples. A *t*-test was used to calculate the significance of the difference in the *p*-value of each compound. The R language package ropls was used to perform OPLS-DA modeling, and 200 permutation tests were performed to verify the reliability of the model. The VIP value of the model was calculated using multiple cross-validation. The method of combining the multiple differences, the *p*-value, and the VIP value of the OPLS-DA model was adopted to screen the differential metabolites. The screening criteria are FC > 1, *p*-value < 0.05, and VIP > 1. The differential metabolites for KEGG pathway enrichment significance were calculated using the hypergeometric distribution test.

## 3. Results

### 3.1. Evaluation of the Bacteriostatic Effect of Tylosin on P. multocida

**Figure 1:**
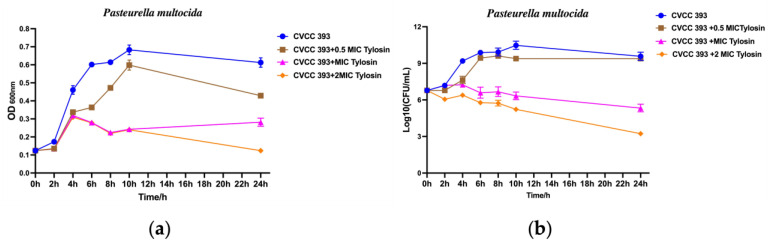
Evaluation of the bacteriostatic activity of tylosin against *P. multocida*. (**a**) Bacteriostatic growth curve. (**b**) Time–kill curve.

In this study, document M26-A described that the MBC is defined as the lowest concentration of antimicrobial agent needed to kill 99.9% of the final inoculum after incubation for 24 h under a standardized set of conditions [17]. The MIC value of tylosin was determined to be 16 μg/mL by the microbroth dilution method. The results of the inhibition curve and the time–kill curve indicated that after 4 h of exposure to MIC and 2 × MIC, bacterial activity decreased (Figure 1). However, under the MIC condition, there was a tendency for the bacteria to recover, while under the 2 × MIC condition, the bacterial load showed a continuous decline, suggesting that tylosin at 2 × MIC concentration could effectively inhibit the growth activity of *P. multocida*. These results indicate that tylosin effectively inhibits the growth of *P. multocida* but does not exert a significant bactericidal effect at the tested concentrations.

### 3.2. Analysis of Differential Metabolites

**Figure 2:**
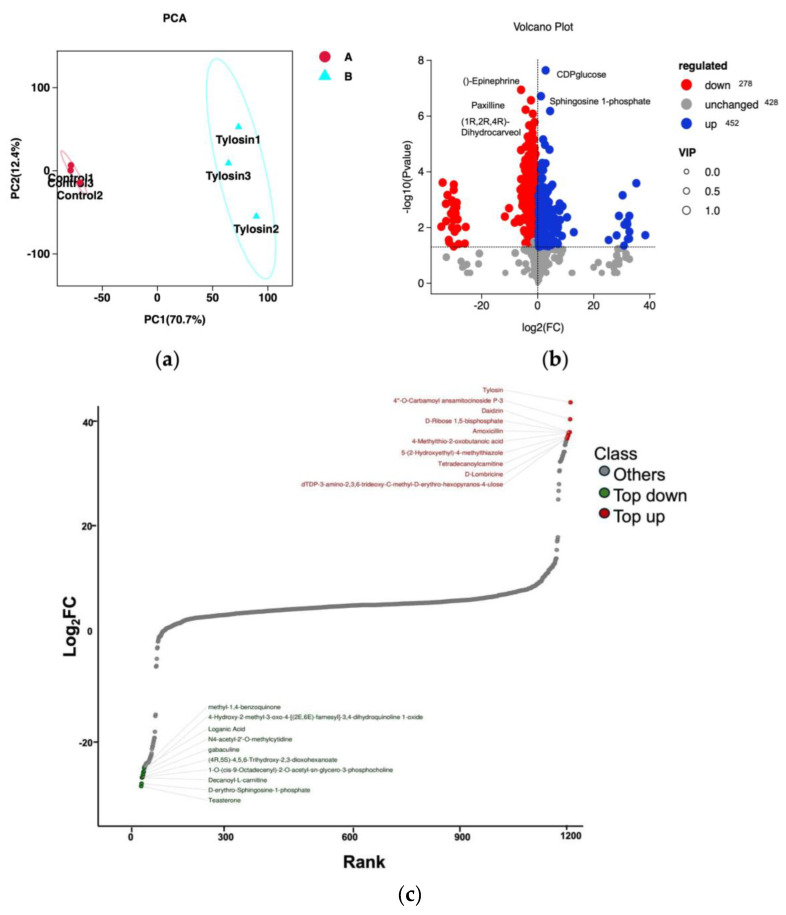
Analysis of differential metabolites. (**a**) Principal component analysis plot for differential grouping: The 95% elliptic confidence interval for the group will be displayed when the number of replicates within the group is greater than or equal to 3. (**b**) Volcano plot of differential metabolites: The blue dots in the figure represent down-regulated differentially expressed metabolites, the red dots represent up-regulated differentially expressed metabolites, and the gray dots represent metabolites that were detected but showed no significant difference. (**c**) Dynamic distribution chart of metabolite content differences: Note: The horizontal axis in the chart represents the cumulative number of substances arranged in ascending order of the difference multiple, and the vertical axis represents the logarithm of the difference multiple to the base of 2.

Through principal component analysis (PCA) of the samples (including quality control samples), the PCA score plot showed a clear separation between the control group (Figure 2a) and the experimental group (Tylosin), indicating that tylosin caused significant metabolic reprogramming. The volcano plot results indicated that a total of 706 metabolites were significantly differentially expressed in the differential metabolite analysis, with 278 up-regulated and 428 down-regulated metabolites (Figure 2b), suggesting that the treatment might affect the consumption or inhibition of related metabolite pathways. Among them, the significantly up-regulated ones were CDP-glucose and sphingosine 1-phosphate, indicating the enhancement of nucleoside sugar metabolism and lipid metabolism pathways.

To more clearly and intuitively display the overall metabolic differences, the fold change (FC) values of the metabolites in the comparison groups were calculated. After calculation, they were arranged in ascending order based on the FC values, and a dynamic distribution map of metabolite content differences was drawn (Figure 2c). The results indicated that the key up-regulated metabolites were mainly antibiotics (tylosin and amoxicillin), deoxynucleoside triphosphates (dTDP-3-amino-2,3,6-trideoxy-C-methyl-D-erythro-hexopyranos-4-ulose), and sugar metabolism intermediates (D-Ribose 1,5-bisphosphate), while growth-related metabolites were down-regulated. This suggests that the defense system of IG *P. multocida* was activated under the action of tylosin, reallocating resources to defensive secondary metabolism (antibiotic synthesis) and cell structure remodeling (nucleoside sugar metabolism) by inhibiting growth and proliferation (down-regulating hormones and terpenoids).

### 3.3. KEGG Functional Annotation and Enrichment Analysis of Differential Metabolites

**Figure 3:**
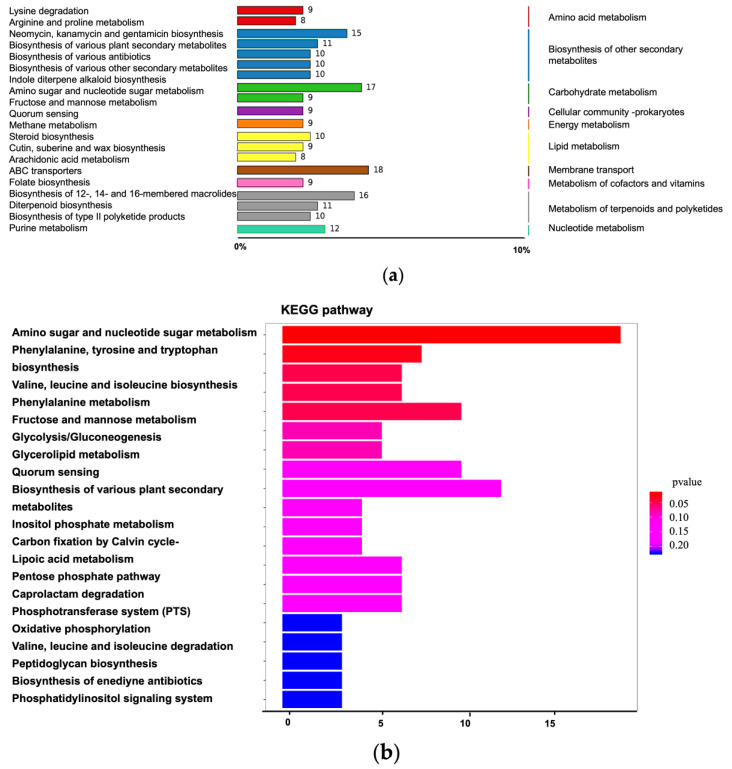
Enrichment analysis of KEGG annotation results of differential metabolites. (**a**) Pathway classification diagram of differential metabolites. (**b**) Bar chart of differential metabolite pathways.

KEGG pathway enrichment analysis of differentially accumulated metabolites was performed using the hypergeometric test implemented in clusterProfiler. As shown in Figure 3a, the top enriched pathways included membrane transport (e.g., ABC transporters; 18 metabolites), carbohydrate metabolism (e.g., amino sugar and nucleotide sugar metabolism; 17 metabolites), and amino acid metabolism—particularly lysine degradation and arginine/proline metabolism (17 metabolites in total). Notably, purine metabolism was significantly enriched with 12 associated metabolites, suggesting heightened nucleotide turnover linked to energy production and nucleic acid biosynthesis. Figure 3b presents the top enriched pathways ranked by statistical significance. In addition to amino acid and nucleotide metabolism, aromatic amino acid metabolism was markedly altered: biosynthetic pathways for phenylalanine, tyrosine, and tryptophan were up-regulated. The valine–leucine–isoleucine biosynthesis pathway was significantly enriched, whereas its corresponding catabolic pathway was suppressed—indicating a net shift toward amino acid accumulation. Glycolysis/gluconeogenesis, quorum sensing, and the pentose phosphate pathway were all enhanced. Conversely, oxidative phosphorylation—the primary mechanism for aerobic ATP generation in bacteria, occurring across the cytoplasmic membrane was significantly down-regulated—implying compromised electron transport chain function. Concurrently, peptidoglycan biosynthesis was reduced, suggesting attenuated cell wall synthesis. Collectively, these findings point to metabolic reprogramming characterized by increased anabolic activity, impaired respiratory metabolism, and diminished structural biosynthesis.

**Figure 4:**
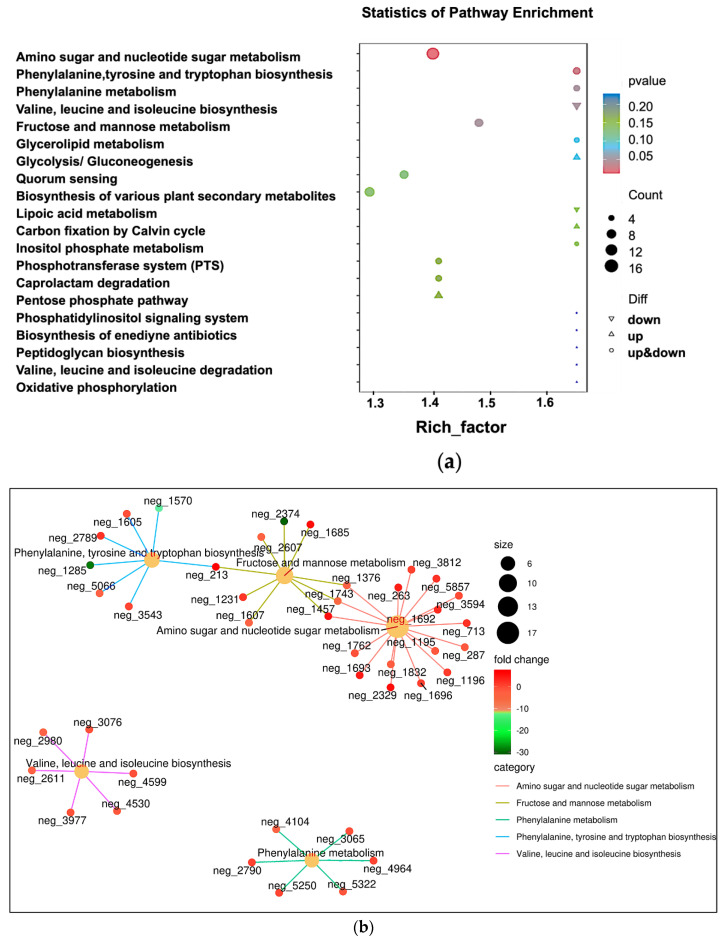
Enrichment analysis of KEGG annotation results of differential metabolites. (**a**) KEGG analysis of differential metabolite enrichment bubble chart represents the ratio of the proportion of differential metabolites annotated to a certain pathway among the differential metabolites and the proportion of metabolites annotated to the pathway among all metabolites. (**b**) KEGG enrichment network diagram of differential metabolites. The light-yellow nodes in the diagram are pathways, and the small nodes connected to them are the specific metabolites annotated to the pathway.

The KEGG enrichment bubble map (Figure 4a) visualized metabolic disorder in bacteria under the dual pressures of membrane damage (decreased oxidative phosphorylation) and cell wall stress (increased peptidoglycan), which eventually shifted to defensive secondary metabolism, such as antibiotic synthesis through phenylalanine metabolism. The pathway enrichment network showed (Figure 4b) that amino sugar and nucleotide sugar metabolism was up-regulated, and about 15 differential metabolites (neg_1692, neg_1195, neg_713, etc.) were connected to form a dense star network; fructose and mannose were metabolized (yellow-green). Phenylalanine–tyrosine–tryptophan biosynthesis (light blue, down-regulated); phenylalanine metabolism (turquoise, down-regulated); branched-chain amino acid biosynthesis (purple, down-regulated) were also affected. In summary, tylosin treatment leads to significant accumulation of multiple amino acids (arginine and proline) in bacteria. This is because protein synthesis is blocked, and amino acids, as raw materials, cannot be consumed. Oxidative phosphorylation is low, while glycolysis is high, suggesting a transition from aerobic respiration to a glycolysis-dependent metabolic mode. The finding that amino acid synthesis is increased and branched-chain amino acid degradation pathways are inhibited provides metabolic evidence for understanding bacterial persistence in chronic infections and for developing therapeutic strategies targeting persisters.

### 3.4. Tylosin Damages the Biofilm of P. multocida

**Figure 5:**
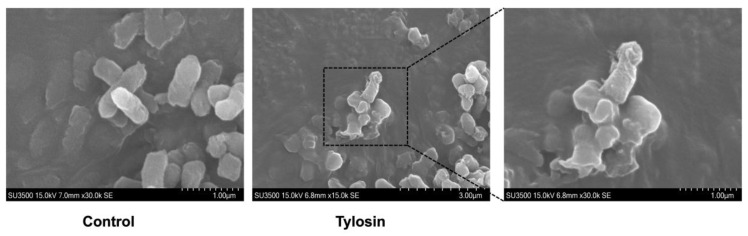
Bacterial ultrastructural analysis (SEM).

To further explore the ability of the compound to destroy bacterial membranes and whether it will damage the ultrastructure of bacteria, morphological changes in *P*. *multocida* after tylosin treatment were observed by scanning electron microscopy. As shown in Figure 5, the blank control group had no obvious effect on the bacterial cell surface, while tylosin treatment showed varying degrees of damage, including irregular bulging or depression, cell surface collapse and lysis, and cell content flow out. These observations indicate that the drug has a significant damaging effect on the bacterial outer membrane, consistent with the metabolomic data. The significant activation of amino sugar and nucleotide sugar metabolism and peptidoglycan biosynthesis pathways indicates that bacteria avoid cell wall defects by activating peptidoglycan biosynthesis; however, the imbalance between synthesis and degradation leads to cell wall perforation.

### 3.5. Tylosin Disrupts the Membrane Functional Integrity of P. multocida

**Figure 6:**
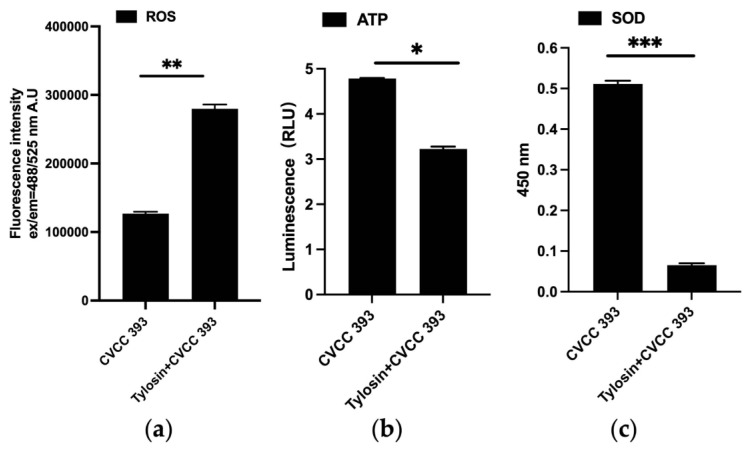
Membrane function determination. (**a**) Determination of ROS in *P. multocida*; (**b**) Determination of ATP in *P. multocida*; (**c**) Determination of SOD in *P. multocida*. * *p* < 0.05， ** *p* < 0.01， *** *p* < 0.001.

To further verify the effect of tylosin on bacterial energy metabolism, as shown in Figure 6, it was found that the ATP content of bacteria in the tylosin treatment group decreased significantly compared with the control group (*p* < 0.05), accompanied by a decrease in SOD activity (*p* < 0.001) and an increase in ROS levels (*p* < 0.01). All these results indicate that tylosin treatment leads to a significant decrease in ATP levels in bacteria, inhibits energy metabolism, promotes the accumulation of ROS, intensifies oxidative stress, disrupts the functional integrity of bacterial biofilms, and affects bacterial energy metabolism, thereby weakening the bacteria’s defense barrier against drug damage. These findings are consistent with the results of metabolomics and electron microscopy, suggesting that after tylosin acts on bacteria, the cell wall is damaged, the cell membrane bulges and ruptures under the action of osmotic pressure, lipid peroxidation occurs, leading to changes in membrane fluidity and increased membrane permeability, resulting in functional energy damage and redox imbalance, and ultimately disrupting the metabolic homeostasis of bacteria.

## 4. Discussion

This study, through non-targeted metabolomics technology, for the first time systematically revealed the antibacterial mechanism of tylosin at higher concentrations against *P. multocida*. The significant reduction in ATP in the drug group may suggest an impaired ability of the bacteria to maintain normal redox homeostasis. During functional verification, the significant decrease in superoxide dismutase (SOD) activity, failure of the defense system, and metabolic disorders jointly led to the accumulation of reactive oxygen species (ROS), which could contribute to oxidative damage to biological macromolecules such as proteins and nucleic acids. This finding is highly consistent with the theory proposed by Lobritz [13], suggesting that antibiotic efficacy may be linked to bacterial cell respiration. The traditional “translation inhibition” view cannot fully explain the rapid bacteriostatic effect of tylosin. Lobritz’s research pointed out that antibiotics induce metabolic disorders and oxidative stress in the respiratory chain, resulting in a lethal burst of ROS. In this study, the stagnation of the TCA cycle may lead to an imbalance in the load of the electron transport chain (ETC), which is hypothesized to drive the accumulation of ROS. The central carbon metabolism stagnation and TCA cycle disorder caused by tylosin essentially trigger a “metabolic collapse” within the bacteria. This phenomenon is consistent with results in *Staphylococcus aureus* [18] and *Pseudomonas aeruginosa* [19]. where metabolic reprogramming, particularly regarding amino acid metabolism (e.g., glutamate and arginine), acts as a potential adaptive response to antimicrobial pressure.

The emergence of antimicrobial resistance (AMR) in bacterial pathogens represents a critical global health threat. While our study does not provide direct genomic evidence of resistance gene transfer, we hypothesize that the metabolic state of *P. multocida* is intimately linked to its survival under antimicrobial pressure. Core metabolic processes—including energy generation, biomass synthesis, cell wall biogenesis, and intercellular signaling—functionally underpin bacterial adaptation and survival under antimicrobial pressure. Consequently, we propose that the observed metabolic fluctuations reflect a coordinated physiological reprogramming of *P. multocida* in response to tylosin, which may theoretically create a favorable environment for bacterial persistence. Notably, our results showed that amino acid and nucleotide metabolism were significantly up-regulated following tylosin treatment. This response might not merely be interpreted as passive metabolite accumulation consequent to halted protein synthesis; rather, it suggests a potential active, adaptive countermeasure. For instance, the specific up-regulation of stress-responsive amino acids, such as arginine and proline, likely functions to mitigate oxidative damage. As demonstrated by Levin [20], which posits that “non-genetic resistance” is grounded in profound physiological determinants, bacterial survival under drug stress is critically dependent on dynamic metabolic fitness rather than static genetic traits. Microbial metabolic signatures hold substantial promise for identifying novel antimicrobial targets and informing mechanism-informed therapeutic strategies [21,22,23]. Nonetheless, we emphasize that these metabolomic profiles are associations; further transcriptomic and functional studies are essential to determine whether this metabolic rewiring directly contributes to the emergence or horizontal transfer of resistance determinants [24].

Tylosin is extensively employed in veterinary medicine for the treatment of respiratory infections caused by *P. multocida*, as well as fowl septicemia, swine enzootic pneumonia, and avian mycoplasmosis [9,25]. As a Gram-negative pathogen, *P. multocida* possesses an outer membrane that constitutes a formidable permeability barrier—this structural feature impedes the intracellular accumulation of tylosin and thereby limits its access to the ribosomal target site. Consequently, poor outer membrane penetration represents a primary determinant of the compound’s suboptimal antibacterial activity and elevated resistance incidence against Gram-negative bacteria, including *P. multocida* and Escherichia coli. Accordingly, strategies aimed at disrupting or circumventing this outer membrane barrier have emerged as a central focus in the rational design of next-generation tylosin derivatives. Clinically, the predominant mechanism of macrolide resistance involves Erm-type rRNA methyltransferase-catalyzed dimethylation of adenine residue A2058 in domain V of 23S rRNA—a modification that sterically hinders macrolide binding. This mechanistic insight has underpinned the structure-guided development of macrolide analogs capable of evading Erm-mediated resistance [26]. Such efforts have culminated in the emergence of macrolide–ketolide hybrids [27]. Metabolomic analyses further substantiate the strategic rationale for C-20 modification of tylosin [28]: enhanced ribosomal affinity—manifested as a reduced dissociation rate—lowers the effective therapeutic concentration required, thereby mitigating TCA cycle disruption and oxidative stress burden in host cells and diminishing associated metabolic toxicity. Critically, this structural optimization strengthens the dual steric and electrostatic blockade of nascent polypeptide chains within the ribosomal exit tunnel: it augments physical obstruction while introducing favorable electrostatic and hydrogen bonding interactions with the ribosome, collectively reinforcing target engagement. As a result, the modified derivative exhibits robust activity against strains harboring prevalent rRNA modifications, offering a sound mechanistic foundation for the development of novel semisynthetic macrolide therapeutics.

While this study provides a comprehensive non-target metabolomic profile of *P. multocida* in response to tylosin, we acknowledge several limitations. First, although our metabolomic analysis provides a comprehensive view of the cellular response to tylosin, it reflects a snapshot of the metabolic state at specific time points under in vitro conditions. Specifically, a limitation of our study is the lack of parallel validation via transcriptomics, proteomics, or enzymatic activity assays to confirm the regulatory pathways proposed herein. Therefore, the observed metabolic shifts should be regarded as potential associations, and future research is warranted to validate these mechanisms. Second, the in vitro nature of this study does not fully capture host immune factors, complex microbial communities, or pharmacokinetics found in vivo. Third, while we observed membrane-related changes, these likely represent secondary physiological consequences of severe metabolic stress rather than a direct bactericidal membrane-disrupting mechanism [29]. Finally, regarding the chemical stability of tylosin, although experimental conditions were controlled, we cannot entirely exclude the possibility that drug degradation products [30,31] may have contributed to the observed metabolic variations. Future studies integrating longitudinal pharmacokinetic data and real-time monitoring of antibiotic concentrations will be necessary to further refine our understanding of these tripartite interactions.

Within the One Health framework, an in-depth understanding of the oxidative stress response network of *P. multocida* is of strategic significance not only for the control of animal diseases but also for the protection of human health. Understanding the vulnerabilities of bacteria in oxidative metabolism under antibiotic attack could lead to the development of precise control techniques that are more environmentally friendly and less likely to induce cross-resistance, thereby reducing the spread of drug resistance genes (ARGs) in the environment and protecting the last line of defense against zoonotic diseases.

## Data Availability

The data presented in this study are available on request from the corresponding author due to (ongoing research based on the same dataset).

## Abbreviations

SEM	Scanning Electron Microscope
MIC	Minimum Inhibitory Concentration
ROS	Reactive Oxygen Species
SOD	Superoxide Dismutase
